# Phylogenomic approaches to determine the zoonotic potential of Shiga toxin-producing *Escherichia coli* (STEC) isolated from Zambian dairy cattle

**DOI:** 10.1038/srep26589

**Published:** 2016-05-25

**Authors:** Geoffrey Mainda, Nadejda Lupolova, Linda Sikakwa, Paul R. Bessell, John B. Muma, Deborah V. Hoyle, Sean P. McAteer, Kirsty Gibbs, Nicola J. Williams, Samuel K. Sheppard, Roberto M. La Ragione, Guido Cordoni, Sally A. Argyle, Sam Wagner, Margo E. Chase-Topping, Timothy J. Dallman, Mark P. Stevens, Barend M. deC. Bronsvoort, David L. Gally

**Affiliations:** 1 Roslin Institute and Royal (Dick) School of Veterinary Studies, Edinburgh, UK; 2 Ministry Livestock and Fisheries, Kabwe, Zambia; 3 University of Zambia, Lusaka, Zambia; 4 University of Liverpool, Liverpool, UK; 5 Swansea University Medical School, Swansea, UK; 6 University of Surrey, Surrey, UK; 7 University of Edinburgh, Edinburgh, UK; 8 Public Health England, London, UK

## Abstract

This study assessed the prevalence and zoonotic potential of Shiga toxin-producing *Escherichia coli* (STEC) sampled from 104 dairy units in the central region of Zambia and compared these with isolates from patients presenting with diarrhoea in the same region. A subset of 297 *E. coli* strains were sequenced allowing *in silico* analyses of phylo- and sero-groups. The majority of the bovine strains clustered in the B1 ‘commensal’ phylogroup (67%) and included a diverse array of serogroups. 11% (41/371) of the isolates from Zambian dairy cattle contained Shiga toxin genes (*stx*) while none (0/73) of the human isolates were positive. While the toxicity of a subset of these isolates was demonstrated, none of the randomly selected STEC belonged to key serogroups associated with human disease and none encoded a type 3 secretion system synonymous with typical enterohaemorrhagic strains. Positive selection for *E. coli* O157:H7 across the farms identified only one positive isolate again indicating this serotype is rare in these animals. In summary, while Stx-encoding *E. coli* strains are common in this dairy population, the majority of these strains are unlikely to cause disease in humans. However, the threat remains of the emergence of strains virulent to humans from this reservoir.

Shiga toxigenic *Escherichia coli* (STEC) are emerging pathogens of public health concern worldwide, including in Europe, North and South America and Asia^1^,^2^. Ruminants, in particular cattle, have been identified as the predominant reservoir of STEC^3^,^4^, indicating that the bacteriophage-encoded Shiga toxins (Stx) are likely to confer an advantage to *E. coli* in these host animals. In Africa there is little information on the epidemiology of STEC in livestock systems and their impact on human health^1^. It is evident that only a subset of STEC are a serious threat to human health, these enterohaemorrhagic *E. coli* (EHEC) are associated with specific serogroups in particular the seven that have been defined as adulterants in beef production in the USA, O157, O26, O111, O45, O145, O103, O121^5^,^6^. Similar serotypes, especially O157 & O26 are also an issue in Europe. Typical EHEC strains can be further characterised by possession of a type 3 secretion system (T3SS) that enables colonisation of the gastrointestinal tract^7^. EHEC infections in humans are associated with diarrhoea and bloody diarrhoea, with the more serious sequelae of kidney and brain damage due to activity of Stx on the microvasculature in these organs^4^,^8^.

The cost of whole genome sequencing (WGS) has drastically reduced and it is now possible to sequence large numbers of isolates and use bioinformatics approaches to extract strain relatedness and gene carriage data. For *E. coli*, strains have classically been assigned into phylogroups that provide a good correlation with commensal versus pathogenic strains^9^. The phylogroups are based on particular combinations of specific genes and can be assigned from the WGS. Whole genome core SNP analysis to define strain relatedness is now commonly used and provides greater resolution than MLST^10^. In addition the serogroup, the O-chain of LPS, can also be inferred from their genetic determinants using WGS. The WGS of any strain collection is therefore a valuable resource allowing relatively rapid comparison of phylogeny and pathogenic potential.

Both small holding and large-scale dairy farming is important to the economic survival of communities in many developing nations, including Zambia^11^,^12^. As such, it is important to understand if practices on these units and their products may represent a threat to human health and where such risks exist suggest possible mitigation measures. A study has recently been carried out to sample *E. coli* strains from cattle across small, medium and large-scale (commercial) dairy farms in central Zambia, with the primary aim of understanding antibiotic use and antimicrobial resistance patterns in this sector^13^.

These isolates have now been further analysed in the present study for *stx* prevalence and any association with the farming system. In addition, *E. coli* isolates from patients with diarrhoea were also screened and sequenced to determine evidence of relationship to the bovine strains.

## Results

### Detection of Shiga toxin alleles (*stx1* and *stx2*) in isolates from Zambian dairy cattle and humans

Eleven percent (41/371) of the bovine *E. coli* isolates were positive for the presence of Shiga toxin genes as defined by detection of appropriately sized PCR amplification products using an established *stx* multiplex assay^14^. Based on this, both *stx1* and *stx2* were detected in 54% (22/41) of the STEC, while 37% (15/41) had *stx2* only and 10% (4/41) had *stx1* only. Using this data, the overall adjusted prevalence of STEC across the different farming scales for the central Zambian study area can be estimated at 6% (95% CI: 2.5–10.2). The adjusted prevalence per farming scale was higher in medium-scale 17.1% (95% CI: 5.9–28.2) and small-scale 10.6% (95% CI: 6.6–14.5) farms when compared to the commercial farms 2.8% (95% CI: 0.3–6.0). Based on these ranges, there is a significant difference in estimated prevalence between the small and commercial scales. Logistic regression indicates that medium- and small-scale farming are significant risk factors for Shiga toxin producing *E. coli* (STEC) with commercial as a reference (Table 1). Out of the 73 *E. coli* isolates from human patients with diarrhoea for which good quality sequence information was generated, no Shiga toxin genes were detected.

As an additional investigation, the enrichment cultures for all the animals (n = 371) were streaked onto sorbitol MacConkey agar plates and any non-sorbitol fermenting colonies tested for O157 agglutination. Only one animal yielded a positive strain (ZB-2213N0194) and this was then added to the study.

### Phylogenetics

In order to understand the genetic backgrounds of the STEC strains isolated in this study, including their potential threat to human health, their relationship to other human disease-associated EHEC were tested by phylogenetic methods. The WGS of 297 of the Zambian isolates (224 bovine and 73 human) were determined. This included 41 STEC, 37 of the 41 defined as *stx*+ by PCR from main study, three STEC strains from a pilot study and the single positively selected *E. coli* O157 strain. These were compared with 262 *E. coli* sequences from human, cattle, avian and canine hosts; one hundred and twenty nine strains in this second collection were human clinical STEC isolates (see Supplementary Table 1).

**Figure 1:**
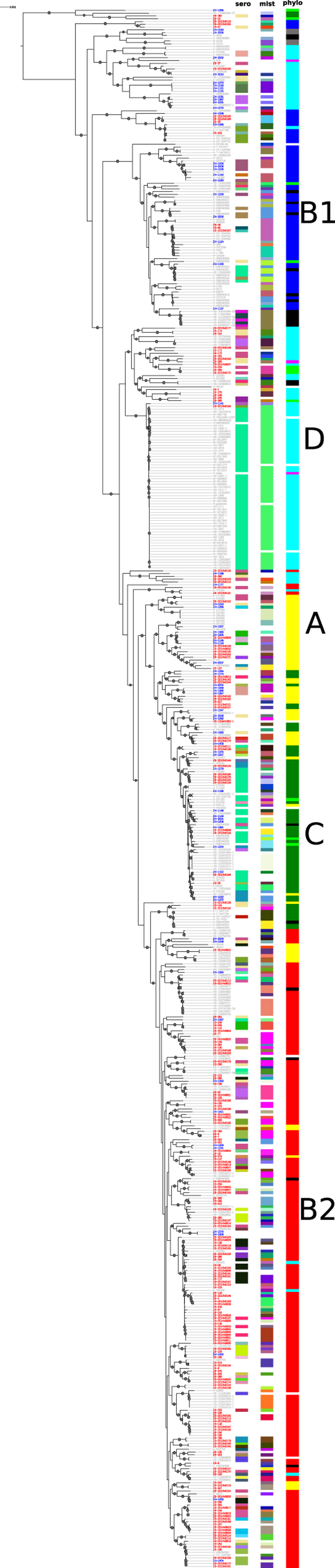
Phylogenetic context of Zambian isolates. The tree depicts the phylogenetic relationship of *E. coli* isolates from Zambia (bovine - red and human - blue) with other *E. coli* isolates (grey). The ML tree is based on core SNPs as described in Materials and Methods. The tree is un-rooted and grey circles on branches represent bootstrap values higher than 80. Vertical columns demonstrate: (1) Diversity of the sequence types (ST) based on MLST analysis where each colour represents a different ST; (2) Diversity of O-serogroups for which each colour represents a different group; (3) Phylogroups: A-yellow, B1-red, B2-blue, C-green, D-turquoise, E-pink, F-grey, cryptic clades-light green. The phylogroups are consistent with core SNP clustering with some minor discordance. White spaces on all columns indicate sequences that were untypable.

Alignment to a reference genome (*E. coli* O157:H7 str. Sakai, RefSeq assembly accession: GCF_000008865) resulted in 715,632 core positions with 68,327 single nucleotide polymorphism (SNPs) across all 559 sequences. A maximum likelihood phylogeny revealed the population structure of the *E. coli* strains (Fig. 1). While there was no clear clustering of the strains based on geographical location or host, there was, as anticipated, good correlation with established *E. coli* phylogroups, with only minor discordance. All possible phylogroups and cryptic clades were identified, however the majority of the *E. coli* strains (97%) were distributed across 5 phylogroups (Fig. 1).

The Zambian bovine strains (n = 224) predominately associated with the B1 ‘commensal’ cluster (67%) with the remainder present as: A (9%); B2 (4%); C (8%); D (9%); other (3%). By contrast, the Zambian human strains (n = 73) had equivalent representation across the 5 main phylogroups: A (22%); B1 (19%); B2 (16%); C (22%); D (16%); other (5%). The Zambian cattle STEC strains were also predominately in the B1 phylogroup (27/41).

### Serotyping

The majority of EHEC strains that are a threat to human health are associated with 7 specific serogroups. A bioinformatic approach was used to serotype the sequenced strains. H typing was possible for 550/559 strains and O-typing for 483/559 (summarized in Supplementary Tables 3 and 4). Failure to detect specific genes in some of the strains was most likely due to assembly issues with short read sequences.

**Figure 2:**
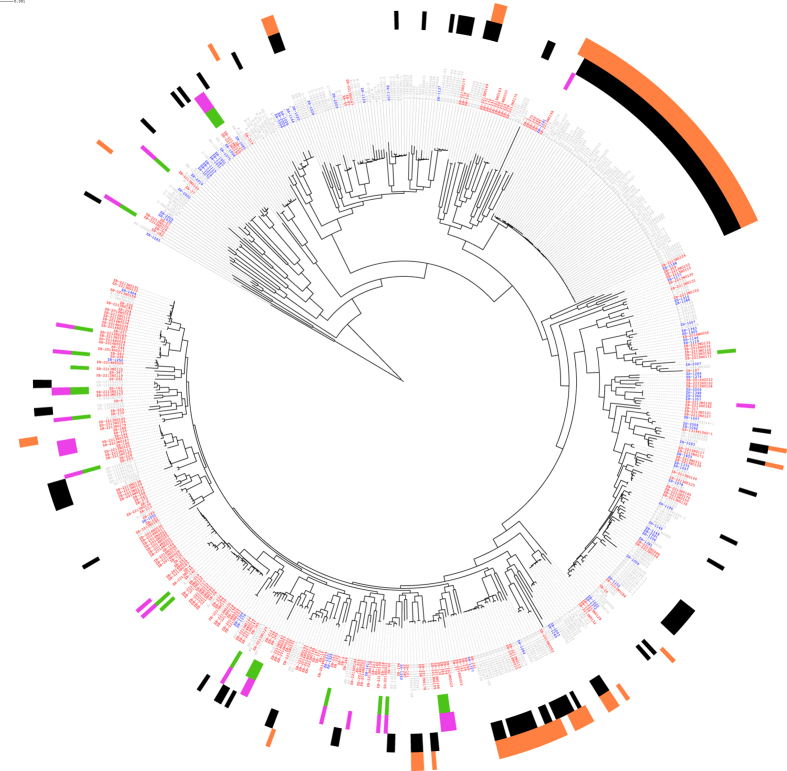
Phylogenetic relationship between STEC. The same ML core SNP tree as in Fig. 1 plotted in a circular manner to depict relationships between Shiga toxin encoding strains. The strain designations are Zambian bovine (red), Zambian human (blue), other *E. coli* (grey). For the Zambian strains, the coloured bars indicate the presence of Stx genes: *stx2* (purple) and *stx1* (green). Black blocks around the tree indicate non-Zambian *E. coli* encoding *stx* (1 or 2). Orange blocks highlight the presence of intimin (*eae*) and *sepL* indicating the possession of a type 3 secretion system. It is apparent that with the exception of one positively selected EHEC O157 (ZB-2213N0194), that the Zambian cattle STEC do not encode this system.

With the exception of the positively selected *E. coli* O157 strain, none of the Zambian bovine STEC strains (0/40) were assigned to any of these seven serogroups. In fact, only 3 strains across the whole set of cattle isolates could be assigned within these serogroups (ZB-244; serogroup O45 and ZB-2213N0112; serogroup O111 and ZB-2213N0194; serogroup O157). Overall, the Zambian strains (cattle and human) exhibited an extensive array of serogroups (Fig. 1 and Supplementary Table 4) and H:O combinations were unique for each strain with little clustering or association with established human clinical isolates (Fig. 2).

### Toxicity analysis of *stx*+ strains

**Figure 3:**
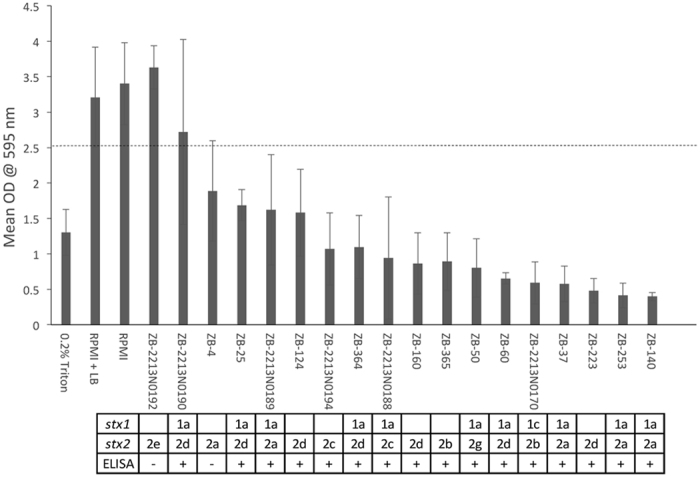
Shiga toxin activity and subtyping. The top panel graph indicates the cytotoxic effect of selected STEC strain supernatants on Vero cells. Increased cell survival results in higher values. 0.2% Triton X-100 was used as a positive control; RPMI + LB and RPMI alone were used as negative controls. Values below the dashed line indicate a cytotoxic effect on the cells. 89% (16/18) of the STEC supernatants tested demonstrated a cytotoxic effect. Supernatants were prepared as described in Materials and Methods. Stx subtypes are shown in the lower panel along with ELISA results for detection of Stx. Isolates with both *stx1a* and *stx2a* are associated with higher toxicity. Sample number ZB-4-stx contains *stx2a* and exhibited cytotoxicity on Vero cells but was negative by ELISA.

To determine if the genotypically positive *stx* strains were able to express Stx, eighteen of the bovine STEC were examined for Vero cell cytotoxicity with and without mitomycin C (MMC) induction. 89% (16/18) of the MMC-induced STEC strains had a cytotoxic effect on Vero cells (Fig. 3). These samples were verified as Stx positive using a commercial ELISA (Fig. 3), with only one strain (4) exhibiting toxicity on Vero cells without any detection of Stx by ELISA.

### Shiga toxin subtyping

Forty one STEC positive strains were included in the WGS analysis (Fig. 2) and from this *stx* alleles could be further subtyped using a published BLAST-based methodology^15^ (Supplementary Table 5). It was evident that the most cytotoxic strains (Fig. 3) were those encoding Stx2a often in combination with Stx1a, in line with studies of cytotoxicity and pathology induced by enterohaemorrhagic strains with different Stx variants^16^,^17^.

### Stx association with type 3 secretion and enteroaggregative virulence factors

Typical enterohaemorrhagic *E. coli* strains are defined by the co-association of *stx* genes with a type 3 secretion system (T3SS)^7^. In the present study, the presence of a T3SS was determined by detection of both *eae* and *sepL*. Based on BLAST analysis, 3.6% (8/224) and 2.7% (2/73) of the Zambian bovine and human isolates respectively may encode a T3SS (Fig. 2). Excluding the positively selected O157 strain, neither intimin (*eae*) nor *sepL* were detected in the bovine STEC. Other non-STEC but intimin positive strains were present within the Zambian strains analysed and some were in close proximity to clinical human STEC strains (Fig. 2). The cattle strains were also checked for the presence of the enteroaggregative *E. coli* (EAEC) adherence factors AggR and AA probe by PCR but all were negative indicating that these dairy cattle are not a common reservoir of enteroaggregative *E. coli* as associated with the atypical EHEC O104 outbreak in Northern Germany in 2011.

## Discussion

Shiga toxins (Stx) can pose a serious threat to human health, and human infections are usually restricted to a subset of serogroups that can express Stx and a type 3 secretion system (T3SS) or other adherence mechanisms that can facilitate colonisation of the human gastrointestinal tract. In this study, *E. coli* isolates were obtained from cattle associated with dairy production in a region of central Zambia. A total of 371 isolates, each from an individual animal, covering 104 farms were tested for the presence of *stx* by PCR. Of these, 41 (11%) were positive. This gives an estimated prevalence (taking into account sampling and the study design effect) of 6% (95% CI: 2.5–10.2). To our knowledge, this is one of the first surveys to systematically analyse the proportion of random *E. coli* from a farm animal source that are positive for *stx* as other studies usually use positive selection methods from animals. Our survey does indicate that STEC are common in these dairy cattle. It was also evident that the small and medium sized production units had a higher prevalence of STEC than the commercial units sampled. This is of interest as it does indicate that management practices potentially influence the selection of STEC. On-going work will investigate these differences including the influence of breed which can differ between the farming scales^13^ and/or diet which can change EHEC O157 prevalence^18^.

Our study also examined 73 *E. coli* strains from human patients with diarrhoea presenting at the University Teaching Hospital (UTH) in Lusaka over the same time period. While the human sample numbers were relatively low, *stx* was not detected, indicating that STEC are unlikely to be common in the local human population. The overall phylogenetic analyses of the strains into phylogroups was as anticipated, with the majority of the Zambian bovine strains being present in the B1 group associated more with commensal strains, although this cluster contains non-O157 EHEC serotypes causing infections in humans that likely originate from cattle. A greater proportion of the human Zambian isolates clustered within the phylogroups associated with human disease, reflecting that the strains were collected from patients with diarrhoea and in some cases the strain may be the etiological agent.

Based on bioinformatic analyses of WGS, there was marked diversity of serotypes in the cattle and human sample populations (Fig. 1 and Supplementary Tables 3 and 4). None of the randomly selected bovine STEC were allocated to a serogroup commonly associated with human EHEC infections. Furthermore, none of these bovine STEC encoded a T3SS based on detection of intimin (*eae*) and *sepL* alleles (Fig. 2), as both genes are present on the locus of enterocyte effacement that encodes the system. An additional study to positively select for *E. coli* O157 isolates from the faecal pat enrichments only identified one positive sample from a farm. Taken together, this study indicates that while STEC are common in the Zambian dairy cattle these strains would not be classified as EHEC and are unlikely to be associated with serious human disease.

While it is encouraging that EHEC strains were extremely rare, many of the supernatants from the STEC strains were cytotoxic and appropriate backgrounds for EHEC emergence are present. As such, we should remain vigilant in case Stx-encoding prophages from this reservoir do emerge in other strain backgrounds that have a higher capacity to cause disease in the human host. Continuing work on factors driving the maintenance of STEC strains in the bovine host will hopefully clarify approaches to reducing the threat from this emerging group of pathogens.

## Methods

### Bovine and human isolates from Zambia

Bovine *E. coli* isolates (n = 371) were collected as part of a previously published study investigating antimicrobial resistance^13^. In addition, a further 81 *E. coli* isolates from cattle were collected as part of a pilot study in 2013 in the same region^13^. Faecal sampling and animal handling of the farm animals was carried out in accordance with the approved guidelines issued by The Roslin Institute Animal Welfare and Ethical Review Body which approved this study^13^. In the main study, 376 dairy cattle from 104 farms representing about 20% of the dairy herds in the study area were randomly sampled and an *E. coli* was isolated from 371 animals (*E. coli* was not isolated from 5 animals) based on growth characteristics on both MacConkey agar and Bile-X-Glucuronide (TBX) plates (Oxoid, UK). Subsequent phylotyping indicated that 97% (361/371) could be allocated to established *E. coli* phylogroups^19^. In terms of subsequent studies the isolates were chosen as follows: From the main study all isolates (n = 371) were tested by the *stx* PCR to allow the prevalence to be estimated. 188 were sequenced but 186 were used in the phylogenetic analyses due to quality issues with 2 sets of reads. The sub-selected strains for sequencing were as follows (those with poor reads removed): (1) All strains showing phenotypic antibiotic resistance in the original study (n = 61)^13^; Strains positive for *stx* by PCR (37/41); the rest (n = 88) were randomly chosen from the remainder. From the pilot study 40 from 81 strains were sequenced including three that were *stx*+ as determined by PCR; 37/40 were included in the phylogenetic study as three had read quality issues. As a separate study, enrichments from all animals (n = 371) were plated onto sorbitol MacConkey and any non-sorbitol fermenting colonies (3 per plate) tested for O157 agglutination. Only one animal yielded a positive isolate (ZB-2213N0194) and this positively selected isolated was then sequenced and added into the phylogenetic analysis. In total there were 224 bovine *E. coli* good quality whole genome sequences that were analysed in this study.

*E. coli* isolates (n = 79) from patients presenting with diarrhoea were collected at Lusaka hospital between 4th December 2014 and 7th January 2015 as part of another project managed by Prof. J.B. Muma and generously supplied for sequencing. Informed consent was obtained from all subjects. Six of the isolate sequences were not analysed due to read quality leaving n = 73 for phylogenetic and virulence determinant analysis. Further strain and sequence details are provided in the Supplementary Table 1.

### DNA extraction

DNA extraction was carried out using either a Wizard Genomic DNA Extraction Kit® or a Qiagen® DNA extraction kit from 1 ml of bacterial culture as defined in the manufacturers’ protocols.

### PCR detection of virulence determinants

All the bovine strains were screened by a published multiplex PCR for Shiga toxin genes and intimin^14^. The strains were also screened with a multiplex PCR for *aggR* and AA probe genes as markers for enteroaggregative *E. coli*^20^. The PCR products were visualised and captured using multi imaging software (Fluorchem HD2) following electrophoresis in 1.5% w/v agarose gel (Agarose, Melford, UK) and staining with Gelred®.

### Verocytotoxicity assays

Established method^21^,^22^, with these minor variations: Single colonies were selected from LB agar plates and suspended in 10 ml of LB broth for 24 h (overnight). 50 μl of overnight culture was added to 5 ml (1:100) of fresh LB broth and incubated for 60 min. Then 20 μl of 5 μg/ml mitomycin C (MMC) was added followed by overnight incubation.

Supernatant samples were screened for the presence or absence of Stx using a commercial ELISA kit (RIDASCREEN® Verotoxin ELISA (C2201), R-Biopharm AG, Darmstad, Germany) according to the manufacturer’s instructions.

### Statistical analysis

The adjustment of prevalence estimates per farming scale and the risk factor analysis were carried out using logistic regression in ‘survey package’^23^ in R software environment version 3.1.1 (http://cran.r-project.org/), *p* < 0.05 values were taken as statistically significant. The statistical analyses and more information on the definition of the different level farming systems were as described previously^13^.

### *E. coli* whole genome sequence analyses

To better understand how the Zambian *E. coli* strains dataset compared with other *E. coli*, the Zambian strain genomic sequences were analysed with a larger strain collection that consisted of 559 *E. coli* genomes, including clinical and commensal isolates from 4 different broad categories of animal and human hosts (Supplementary Table 1). New short read sequence files have been uploaded to European Nucleotide Archive under the study accession number: PRJEB11782, PRJEB11950, PRJEB11956. Some genome sequences from the Zambian strain sets were removed due to poor read and/or assembly quality, resulting in 297 Zambian genome sequences (224 bovine and 73 human) available for analysis.

### Sequencing analysis

All reads were generated by Illumina 1.9 paired-end read sequencing with read lengths from 36 to 251 bp. FASTQC^24^ was used for quality assessment and where necessary trimming was done with cutadapt^25^. Short reads were aligned to a reference *E. coli* O157:H7 str. Sakai (RefSeq assembly accession: GCF_000008865) by combining BWA^26^, SAMtools and SnpEff^27^ in a custom-made python script. The consensus sequence for each alignment of 5,590,092 bp was produced using the majority rule.

Consensus sequences for each alignment were concatenated into one multifasta file that were then parsed to find core positions. Multifasta files of concatenated core nucleotides for each strain were used for recombination analysis with GUBBINS^28^. The recombinatorial regions were removed from the final alignment. The final alignment was then used to construct a Maximum Likelihood (ML) tree with RAxML^29^ under a GAMMA model of heterogeneity with 100 bootstrap replicates (BS). The trees were visualised with ITOL^30^.

An established phylotyping scheme^31^ was used as a starting point to develop a programme that assigned each strain to one of the 4 possible phylogroups (A, B1, B2, D) based on the presence or absence of one of 3 genes *chuA*, *yjaA*, *arpA* and one genetic fragment *TspE4.C2*. To further distinguish between groups and assign strains to an additional 4 phylogroups (C, E, F or cryptic clades), it was necessary to check for the presence of a fifth gene *trpA* and for the presence of specific alleles for the above genes. *arpA* alleles were used to distinguish between phylogroups D and E based on specific primer sequences described in^31^.

To establish gene presence or absence a database that includes all sequences from the collection were built with BLAST+^15^. Query gene’s sequences of intimin, *sepL*, *chuA*, *yjaA*, *arpA*, *trpA*, genetic fragment TspE4.C2 were downloaded from the NCBI website. Gene identifiers are presented in the (Supplementary Table 2). Query Shiga toxin sequences identified in^32^ also were downloaded from the NCBI website. Gene’s presence were established based on a E-value = 0 and similarity match at >90% coverage of the query sequence. For Shiga toxins blast results were filtered based on bit score above 1000, if multiple contigs were involved only the highest result was kept.

Serogroups were identified based on presence of one or several alleles from the following genes: for O-typing - *wzx*, *wzy wzm* and *wzt*; for H-typing the flagellin genes *fliC*, *flkA*, *fllA*, *flmA* and *flnA*. Databases were provided by Dr Flemming Scheutz and colleges^32^. Multi locus sequence type were identified using SRST2 software^33^.

## Additional Information

**Accession codes**: Short read sequence files have been uploaded to European Nucleotide Archive under the study accession number: PRJEB11782, PRJEB11950, PRJEB11956.

**How to cite this article**: Mainda, G. *et al.* Phylogenomic approaches to determine the zoonotic potential of Shiga toxin-producing *Escherichia coli* (STEC) isolated from Zambian dairy cattle. *Sci. Rep.* **6**, 26589; doi: 10.1038/srep26589 (2016).

## Supplementary Material

Supplementary Information

## Figures and Tables

**Table 1:** Farming type STEC risk analysis.

Farm type	Estimate	95% CI	P
Commercial	1	–	–
Medium	7.05	1.76–28.28	0.007
Small	4.05	1.20–13.64	0.002

## References

[b1] Islam M. Z. *et al.* Regional variation in the prevalence of *E. coli* O157 in cattle: A meta-analysis and meta-regression. PloS One 9 10.1371/journal.pone.0093299 (2014). 10.1371/journal.pone.0093299 PMC3972218 24691253

[b2] Chase-Topping M. E. *et al.* Pathogenic potential to humans of bovine *Escherichia coli* O26, Scotland. Emerging Infectious Diseases 18, 439–448 (2012). 22377426 10.3201/eid1803.111236 PMC3309639

[b3] Pruimboom-Brees I. M. *et al.* Cattle lack vascular receptors for *Escherichia coli* O157: H7 Shiga toxins. Proceedings of the National Academy of Sciences 97, 10325–10329 (2000). 10.1073/pnas.190329997 PMC27023 10973498

[b4] Karch H. *et al.* In Zoonoses-Infections Affecting Humans and Animals 235–248 (Springer, 2015). URL: http://link.springer.com/chapter/10.1007/978-94-017-9457-2_9#page-1. (Date of access: 15/11/2015).

[b5] Pearce M. *et al.* Prevalence and virulence factors of *Escherichia coli* serogroups O26, O103, O111, and O145 shed by cattle in Scotland. Applied and Environmental Microbiology 72, 653–659 (2006). 16391103 10.1128/AEM.72.1.653-659.2006 PMC1352218

[b6] Friesema I. *et al.* Emergence of *Escherichia coli* encoding Shiga toxin 2f in human Shiga toxin-producing *E. coli* (STEC) infections in the Netherlands, January 2008 to December 2011. Euro Surveill 19, 26–32 (2014). 24821123

[b7] Kaper J. B., Nataro J. P. & Mobley H. L. Pathogenic *Escherichia coli*. Nature Reviews Microbiology 2, 123–140 (2004). 15040260 10.1038/nrmicro818

[b8] Fruth A., Prager R., Tietze E., Rabsch W. & Flieger A. Molecular epidemiological view on Shiga toxin-producing *Escherichia coli* causing human disease in Germany: Diversity, prevalence, and outbreaks. International Journal of Medical Microbiology (2015). URL: http://www.sciencedirect.com/science/article/pii/S1438422115000831. (Date of access: 15/11/ 2015). 10.1016/j.ijmm.2015.08.020 26372529

[b9] Clermont O., Gordon D. & Denamur E. A guide to the various phylogenetic classification schemes for *Escherichia coli* and the correspondence among schemes. *Microbiology*, mic. 0.000063 (2015). URL: http://mic.microbiologyresearch.org/content/journal/micro/10.1099/mic.0.000063. (Date of access: 15/11/2015). 10.1099/mic.0.000063 25714816

[b10] Hasman H. *et al.* Rapid whole genome sequencing for the detection and characterization of microorganisms directly from clinical samples. Journal of Clinical Microbiology, JCM. 02452–02413 (2013). 10.1128/JCM.02452-13 PMC3911411 24172157

[b11] Mulemba H. The Livestock Sector in Zambia and Rising Food Prices Country Briefing–Zambia. International Institute for Sustainable Development, Manitoba, Canada (2009). http://www.iisd.org/sites/default/files/pdf/ag_scenarios_south_africa_zambia.pdf. (Date of access: 15/11/ 2015).

[b12] Mumba C. Economic Analysis of the Viability of Small holder Dairy Farming in Zambia, The University of Zambia, (2012). URL: http://dspace.unza.zm:8080/xmlui/handle/123456789/1804. (Date of access: 15/11/2015).

[b13] Mainda G. *et al.* Prevalence and patterns of antimicrobial resistance among *Escherichia coli* isolated from Zambian dairy cattle across different production systems. Scientific Reports 5 (2015). 10.1038/srep12439 PMC4515737 26211388

[b14] Paton A. W. & Paton J. C. Detection and Characterization of Shiga Toxigenic*Escherichia coli* by Using Multiplex PCR Assays forstx 1, stx 2, eaeA, Enterohemorrhagic E. coli hlyA, rfb O111, andrfb O157. Journal of Clinical Microbiology 36, 598–602 (1998). 9466788 10.1128/jcm.36.2.598-602.1998 PMC104589

[b15] Camacho C. *et al.* BLAST+: architecture and applications. BMC bioinformatics 10, 421 (2009). 20003500 10.1186/1471-2105-10-421 PMC2803857

[b16] Zweifel C., Cernela N. & Stephan R. Detection of the Emerging Shiga Toxin-Producing *Escherichia coli* O26: H11/H-ST29 in Human Patients and Healthy Cattle in Switzerland. Applied and Environmental Microbiology, AEM. 01728–01713 (2013). http://aem.asm.org/content/early/2013/06/24/AEM.01728-13.short. (Date of access: 15/11/2015). 10.1128/AEM.01728-13 PMC3753966 23811503

[b17] Amézquita-López B. *et al.* Detection of Shiga Toxin Variants, Virulence Genes and the Relationship to Cytotoxicity of Shiga Toxin-Producing *Escherichia coli* (STEC) from Domestic Farm Animals. in *Meeting Abstract.* URL: http://sistemanodalsinaloa.gob.mx/archivoscomprobatorios/_14_resumeneventoscientificos/694.pdf. (Date of access: 17/12/2015).

[b18] Fox J., Depenbusch B., Drouillard J. & Nagaraja T. Dry-rolled or steam-flaked grain-based diets and fecal shedding of O157 in feedlot cattle. Journal of Animal Science 85, 1207–1212 (2007). 17224458 10.2527/jas.2006-079

[b19] Clermont O., Bonacorsi S. & Bingen E. Rapid and simple determination of the*Escherichia coli* phylogenetic group. Applied and Environmental Microbiology 66, 4555–4558 (2000). 11010916 10.1128/aem.66.10.4555-4558.2000 PMC92342

[b20] Cerna J. F., Nataro J. P. & Estrada-Garcia T. Multiplex PCR for detection of three plasmid-borne genes of enteroaggregative *Escherichia coli* strains. Journal of Clinical Microbiology 41, 2138–2140 (2003). 12734261 10.1128/JCM.41.5.2138-2140.2003 PMC154749

[b21] Padhye V., Zhao T. & Doyle M. Production and characterisation of monoclonal antibodies to Verotoxins 1 and 2 from *Escherichia coli* of serotype O157: H7. Journal of Medical Microbiology 30, 219–226 (1989). 2479749 10.1099/00222615-30-3-219

[b22] Krüger A., Lucchesi P. M. & Parma A. E. Verotoxins in bovine and meat verotoxin-producing *Escherichia coli* isolates: type, number of variants, and relationship to cytotoxicity. Applied and Environmental Microbiology 77, 73–79 (2011). 21037301 10.1128/AEM.01445-10 PMC3019693

[b23] Lumley T. Analysis of complex survey samples. Journal of Statistical Software 9, 1–19 (2004).

[b24] Andrews S. (2011). URL: http://www.bioinformatics.babraham.ac.uk/projects/fastqc/. (Date of access: 20/11/2015).

[b25] Martin M. Cutadapt removes adapter sequences from high-throughput sequencing reads. EMBnet. Journal 17, pp. 10–12 (2011).

[b26] Li H. & Durbin R. Fast and accurate short read alignment with Burrows–Wheeler transform. Bioinformatics 25, 1754–1760 (2009). 19451168 10.1093/bioinformatics/btp324 PMC2705234

[b27] Li H. *et al.* The sequence alignment/map format and SAMtools. Bioinformatics 25, 2078–2079 (2009). 19505943 10.1093/bioinformatics/btp352 PMC2723002

[b28] Croucher N. J. *et al.* Rapid phylogenetic analysis of large samples of recombinant bacterial whole genome sequences using Gubbins. Nucleic Acids Research, gku1196 (2014). URL: http://nar.oxfordjournals.org/content/early/2014/11/20/nar.gku1196.short. (Date of access: 15/11/2015). 10.1093/nar/gku1196 PMC4330336 25414349

[b29] Stamatakis A. RAxML version 8: a tool for phylogenetic analysis and post-analysis of large phylogenies. Bioinformatics 30, 1312–1313 (2014). 24451623 10.1093/bioinformatics/btu033 PMC3998144

[b30] Letunic I. & Bork P. Interactive Tree Of Life (iTOL): an online tool for phylogenetic tree display and annotation. Bioinformatics 23, 127–128 (2007). 17050570 10.1093/bioinformatics/btl529

[b31] Clermont O., Christenson J. K., Denamur E. & Gordon D. M. The Clermont *Escherichia coli* phylo‐typing method revisited: improvement of specificity and detection of new phylo‐groups. Environmental Microbiology Reports 5, 58–65 (2013). 23757131 10.1111/1758-2229.12019

[b32] Scheutz F. *et al.* Multicenter evaluation of a sequence-based protocol for subtyping Shiga toxins and standardizing Stx nomenclature. Journal of Clinical Microbiology 50, 2951–2963 (2012). 22760050 10.1128/JCM.00860-12 PMC3421821

[b33] Inouye M. *et al.* SRST2: Rapid genomic surveillance for public health and hospital microbiology labs. Genome Med 6, 90 (2014). 25422674 10.1186/s13073-014-0090-6 PMC4237778

