# Supplementary material for: Phylogenomic approaches to determine the zoonotic potential of Shiga toxin-producing Escherichia coli (STEC) isolated from Zambian dairy cattle
Source: Sci Rep. 2016 May 25;6:26589. doi: 10.1038/srep26589 (PMC4879551; doi:10.1038/srep26589)
Supplement: Supplementary Information [file srep26589-s1.pdf]

Supplementary Information for:

Phylogenomic approaches to determine the zoonotic potential of Shiga toxin-producing Escherichia coli (STEC) isolated from Zambian dairy cattle.

Geoffrey Mainda
Nadejda Lupolova
Linda Sikakwa
Paul R. Bessell
John B. Muma
Deborah Hoyle
Sean P. McAteer
Kirsty Gibbs
Nicola J. Williams
Samuel K. Sheppard
Roberto La Ragione
Guido Cordoni
Sally A. Argyle
Sam Wagner
Margo E. Chase-Topping
Timothy J. Dallman
Mark P. Stevens
Barend M. deC. Bronsvoort
David L. Gally

Supplementary Table 1: Isolate sequences used in the study

**Host**	**Source (country)/**<br><br>**Name prefix**	**Number/**	**Remark and source publication if relevant**
**Avian**	Chicken (various)<br><br>A-<br><br>A-CH-	39	This study, two collections of avian isolates. (i) Disease associated isolates from the UK, Italy and Germany, courtesy of Zoetis Animal Health (ii) *E. coli* strains from the GIT of healthy birds (UK). Sequences released to public 31 January 2016 http://www.ebi.ac.uk/ena/data/view/PRJEB11956
	Turkey (Germany and Italy)<br><br>A-T-	6	Isolates from diseased birds in Germany and Italy, courtesy of Zoetis Animal Health
	Duck (Germany)<br><br>A-DK	4	Isolates from diseased birds courtesy of Zoetis Animal Health
**Bovine**	Cattle (Zambian)<br><br>ZB-	224	Isolates from cattle fecal sampling in central Zambia (1). Sequences released to public 31 January 2016 http://www.ebi.ac.uk/ena/data/view/PRJEB11782
	Cattle (UK)<br><br>W-	20	A subset of *E. coli* O157 strains isolated from UK cattle (2)
**Canine**	Canine (UK)<br><br>C-	18	Multi-drug resistant strains isolated from dogs at the Edinburgh University Veterinary School (3) Sequences release to public 31 January 2016 http://www.ebi.ac.uk/ena/data/view/PRJEB11950
	Community (UK)<br><br>C-	19	A subset of strains associated with community-acquired canine UTI (3) Sequences release to public 31 January 2016 http://www.ebi.ac.uk/ena/data/view/PRJEB11950
**Human**	Human (UK)<br><br>HO-<br><br>HS-	122	UK STEC strains as published (4, 5, 6) or this study
	Human (Zambia)<br><br>ZH-	73	Isolated from patients exhibiting symptoms of diarrhea (this study). Sequences release to public 31 January 2016 http://www.ebi.ac.uk/ena/data/view/PRJEB11782
	Shigella isolates (UK)<br><br>R-	3	NCBI:<br><br>Ss046, Sb227, Sd197
	Reference genomes (various)<br><br>R-	31	NCBI:<br><br>H10407, REL606, HS, IAI1, E24377A, 55989, SE11, TW14359-O157, Sakai, Godstone, SMS, IAI39, CE10, 42, UMNO26, E2348-69, SE15, JJ1886, NA114, 536, S88, IHE3034, PMV, UTI89, UM146, LF82, 857C, 83972, CFT073, W3110, MG1655

Table 1 References:

1. Mainda G, Bessell PB, Muma JB, McAteer SP, Chase-Topping ME, Gibbons J, Stevens MP, Gally DL, Bronsvoort BM. 2015. Prevalence and patterns of antimicrobial resistance among *Escherichia coli* isolated from Zambian dairy cattle across different production systems. Scientific reports. 2015;5.

2. Dallman TG, Ashton PM, Byrne L, Perry NT, PetrovskaL, Ellis R, Allison L, Hanson M, Holmes A, Gunn GJ, Chase-Topping, Woolhouse MEJ, Grant KA, Gally DL, Wain J, Jenkins C. 2015. Applying phylogenomics to understand the emergence of Shiga-toxin-producing *Escherichia coli* O157:H7 strains causing severe human disease in the UK. Microbial Genomics, 10.1099/mgen.0.000029.

3. Wagner S, Gally DL, Argyle SA. 2014. Multidrug-resistant *Escherichia coli* from canine urinary tract infections tend to have commensal phylotypes, lower prevalence of virulence determinants and ampC-replicons. Vet Microbiol. 169:171-8. doi: 10.1016/j.vetmic.2014.01.003.

4. Dallman TJ, Byrne L, Ashton PM, Cowley LA, Perry NT, Adak G, Petrovska L, Ellis RJ, Elson R, Underwood A, Green J, Hanage WP, Jenkins C, Grant K, Wain J. 2015. Whole-genome sequencing for national surveillance of Shiga toxin-producing *Escherichia coli* O157. Clin Infect Dis. 61:305-12. doi: 10.1093/cid/civ318.

5. Dallman TJ, Chattaway MA, Cowley LA, Doumith M, Tewolde R, Wooldridge DJ, Underwood A, Ready D, Wain J, Foster K, Grant KA, Jenkins C. 2014. An investigation of the diversity of strains of enteroaggregative *Escherichia coli* isolated from cases associated with a large multi-pathogen foodborne outbreak in the UK. PLoS One 9(5):e98103. doi: 10.1371/journal.pone.0098103.

Supplementary Table 2. Gene Identifiers

Gene	GI	Position
arpA	556503834	4222487-4220301
chuA	15829254	4391446-4389464
trpA	556503834	1317222-1316416
TspE4.C2	7330942	Not applicable
yjaA	556503834	4213234-4213617
SepL	NC_002695.1	4593776 - 4594831
eae	NC_002695.1	4596458 - 4599262

Supplementary Table 3: Most frequent H-types defined from whole genome sequence analysis

Collection (all including Zambian)		Zambian only	
H type (44 types identified)	Number of strains 550	H type (42 types identified)	Number of strains 297
H7	242	H21	38
H4	47	H8	26
H21	47	H7	25
H8	31	H4	22
H10	23	H10	16

Supplementary Table 4: Most frequent O types assigned from whole genome analysis

Collection (all including Zambian)		Zambian only	
O type (129 types identified)	Number of strains 483	O type (102 types identified)	Number of strains 235
O157	57	O8	17
O8	20	O25	9
O6	16	O102	9
O25	13	O6	6
O117	13	O150	6

Supplementary Table 5: Shiga toxin subtypes of bovine Zambian STEC

Shiga toxin	Subtype	Number of isolates	%
**stx1 only**	sxt1a	4	9.76
**stx2 only**	stx2a	3	7.32
	stx2b	1	2.44
	stx2d	7	17.07
	stx2e	1	2.44
	stx2c	2	4.88
**Stx1 and stx2**	stx1a, stx2d	9	21.95
	stx1a,stx2a	10	24.39
	stx1a,stx2g	1	2.44
	stx1c, stx2b	1	2.44
	stx1a,stx2c	2	4.88
